# Type of gesture, valence, and gaze modulate the influence of gestures on observer's behaviors

**DOI:** 10.3389/fnhum.2013.00542

**Published:** 2013-09-05

**Authors:** Elisa De Stefani, Alessandro Innocenti, Claudio Secchi, Veronica Papa, Maurizio Gentilucci

**Affiliations:** ^1^Department of Neuroscience, University of ParmaParma, Italy; ^2^Health Sciences Department, Milan UniversityMilano, Italy; ^3^Rete Multidisciplinare Tecnologica, Istituto Italiano di Tecnologia, University of ParmaParma, Italy

**Keywords:** request gestures, symbolic gestures, gaze, gesture valence, reaching-grasping, arm human kinematics

## Abstract

The present kinematic study aimed at determining whether the observation of arm/hand gestures performed by conspecifics affected an action apparently unrelated to the gesture (i.e., reaching-grasping). In 3 experiments we examined the influence of different gestures on action kinematics. We also analyzed the effects of words corresponding in meaning to the gestures, on the same action. In Experiment 1, the type of gesture, valence and actor's gaze were the investigated variables Participants executed the action of reaching-grasping after discriminating whether the gestures produced by a conspecific were meaningful or not. The meaningful gestures were request or symbolic and their valence was positive or negative. They were presented by the conspecific either blindfolded or not. In control Experiment 2 we searched for effects of the sole gaze, and, in Experiment 3, the effects of the same characteristics of words corresponding in meaning to the gestures and visually presented by the conspecific. Type of gesture, valence, and gaze influenced the actual action kinematics; these effects were similar, but not the same as those induced by words. We proposed that the signal activated a response which made the actual action faster for negative valence of gesture, whereas for request signals and available gaze, the response interfered with the actual action more than symbolic signals and not available gaze. Finally, we proposed the existence of a common circuit involved in the comprehension of gestures and words and in the activation of consequent responses to them.

## Introduction

Gesture is a universal feature of human communication. In every culture speakers produce gestures, although their extent and typology can vary. Some types of gestures, called gesticulations, necessarily accompany speech and are used to add information to the sentence. Other types of gestures are usually produced in isolation even though they can be accompanied by the corresponding words. They transmit the same message as the words even though some aspects are different. These are transferred to word information when the two signals, gestural and verbal, are simultaneously produced (Bernardis and Gentilucci, 2006; Gentilucci et al., 2006; Barbieri et al., 2009). Thus, gesture and speech can be functionally integrated with each other (Gentilucci and Dalla Volta, 2008; Gentilucci et al., 2008).

There are two main classes of gestures which can be produced in isolation and known as conveying social intent: symbolic (or emblematic) and request gestures. Both of them apparently have no relation with pantomimes of transitive actions (i.e., actions directed to an object). The distinction between the two types of gesture is that the former are language-like and communicative of semantic content (Kendon, 1988, 2004; McNeill, 1992, 2000). The latter, besides conveying communicative intent, are more interactive, being used to initiate, maintain, regulate, or terminate various types of interaction. They are also called instrumental because they are designed to immediately influence the behavior of another individual (Barten, 1979).

If the gestures have interactive characteristics, then their production could influence the behavior of an observer. Concerning request gestures, this was observed in previous studies where the authors found that the kinematics of reaching-grasping and placing and those of reaching-grasping and lifting were altered by the request gestures “give-me-in-the-hand” (Sartori et al., 2009), “feed-me” (Ferri et al., 2011) and “pour” (Innocenti et al., 2012). The fact that gestures understanding and responses preparing are interlaced processes also comes from a fMRI study (Newman-Norlund et al., 2007). These authors found that the same frontal areas are involved in both understanding gesture meaning and preparing responses to it.

Thus, the first characteristic providing a social intent that can differentially influence the behavior of an observer is the type of gesture. Indeed, symbolic gestures convey greater semantic content (Kendon, 1988, 2004; McNeill, 1992, 2000), whereas request gestures are more interactive (Barten, 1979). Thus, we expected interference of the type of gesture with the actual action, which, however, could be stronger for request than symbolic gestures. The interference could induce movement slowing down or veering away from the gesturing conspecific (Tipper et al., 1991, 1997; De Stefani et al., 2012; Innocenti et al., 2012).

The second interactive characteristic of gesture is its valence. The positive valence could induce approaching in order to cooperate, whereas the negative valence could induce an attitude to compete. The latter could occur when avoiding the gesturing conspecific or blocking a movement in response to negative gesture was not allowed by the task (e.g., the task necessarily required an action directed toward the gesturing conspecific). If the valence of the gesture was implicitly understood and influenced the behavior of an observer, positive and negative gesture could differently affect the action kinematics. Specifically, negative valence (inducing competition) should make the movement faster when compared to positive valence (inducing cooperation, Georgiou et al., 2007). This hypothesis is supported by previous kinematic data (Ferri et al., 2010) showing that negative expressions of a conspecific such as disgust, made faster feeding movements toward him, as compared to positive expressions.

Previous studies (Sartori et al., 2009; Ferri et al., 2011) tested the availability of gaze: the effectiveness of a request gesture produced by a non-blindfolded receiver was greater than that of a blindfolded receiver. The gaze direction is also a strong index of the intention to initiate a relation: indeed, the direct or averted gaze expresses an intention to interact or not, respectively (Allison et al., 2000; George and Conty, 2008; Senju and Johnson, 2009). Innocenti et al. (2012) found that the direct gaze influenced the observer/agent's kinematics of a sequence constituted by reaching-grasping and lifting a glass. Thus, we searched for effects of not available gaze and direct gaze on action kinematics. We hypothesized that types of gesture and/or valence could differently act on the observer's behaviors according to conspecific's gaze. We expected an interference effect due to gaze and/or the increase in the effects of the other two factors (valence and type of gesture) when the conspecific's gaze was direct.

The possibility that the characteristics of gestures affect actions was verified in Experiment 1. In control Experiment 2 we verified whether the direct gaze alone as compared to not available gaze of a non-gesturing conspecific was sufficient to influence the actual action.

Previous studies (for reviews see Gentilucci and Dalla Volta, 2008; Gentilucci et al., 2008; Gentilucci and Campione, 2011) have shown that gestures and speech are functionally related because aspects of the gesture meaning can be transferred to speech which in turn can modify the gesture kinematics (Bernardis and Gentilucci, 2006; Barbieri et al., 2009). Support to this idea also comes from data by Willems et al. (2007), who investigated the neural network involved in the integration of semantic information from speech with iconic gestures using fMRI. The results showed that premotor areas (BA 6), Broca's area and adjacent cortex (left inferior frontal cortex, BA 45/47) were specifically modulated by integration of gesture information with a language context. ERP studies conducted on gesture–speech integration found that the N400 component is modulated by congruence/incongruence between gestures and verbal expressions (Ozyürek et al., 2007; Wu and Coulson, 2007; Fabbri-Destro et al., unpublished results). As a consequence of these results, we raised the problem of whether the same characteristics of the verbal and gestural message can influence the behavior of an observer and, in affirmative case, in the same or different way. In Experiment 3, words instead of the corresponding-in-meaning gestures were visually presented and effects of the words on the action were searched for.

In previous studies (Sartori et al., 2009; Ferri et al., 2011; Innocenti et al., 2012) the gestures were presented by an individual physically present who theoretically could intervene during execution of the actual actions. Consequently, the observed effects could be due to observer greater visual control of the gesturing conspecific (who could actually interact with the observer), rather than to the message transmitted by the gesture. In the present study this problem was avoided by presenting stimuli which were pictures of a conspecific who had assumed the final posture of a gesture. The presentation of postures also prevented automatic imitation of the gesture kinematics (Campione and Gentilucci, 2011).

## Methods

### Experiment 1

#### Participants

Fourteen right-handed (Oldfield, 1971), naïve volunteers (3 females and 11 males, age 22–25 years) participated in the experiment. All of them were Italian native speakers. The Ethics Committee of the Medical Faculty at the University of Parma approved all the study, which was carried out according to the declaration of Helsinki.

#### Apparatus, stimuli, and procedure

**Figure 1:**
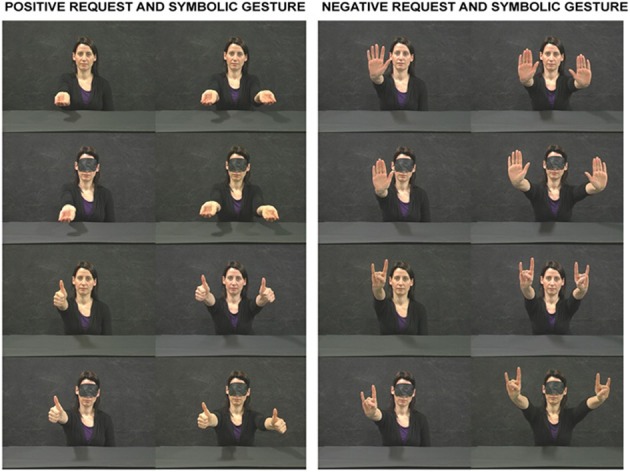
**Meaningful gestures produced by a conspecific and presented in Experiment 1.** The conspecific could be blindfolded or not. The upper two rows show the final posture of request gestures and the lower two rows show the final posture of symbolic gestures. The two columns on the left show gestures with positive valence whereas the two columns on the right show gestures with negative valence.

The participants sat comfortably in front of a table on which they placed their right hand with the thumb and index finger in pinch position (Starting Position, SP). SP was along the participants' mid-sagittal plane and was 30 cm distant from their chest. The monitor of a PC (19 inches SONY LCD) was placed on the table plane, 70 cm distant from the participant's forehead. The monitor was set at a spatial resolution of 1024 × 768 pixels and at a temporal resolution of 60 Hz. The stimuli presented in the monitor consisted of static postures produced by a conspecific and representing either meaningful or meaningless gestures. The meaningful gestures could have positive or negative valence and could be request (“give-me-in-the-hand” and “stop”), or symbolic (“well-done” and “horns”); they were assumed by the conspecific either blindfolded or directing his gaze straight ahead (i.e., toward the participant, Figure 1). All gestures could be postures engaging either the conspecific's right hand (unimanual gesture) or both of the conspecific's hands (bimanual gesture). Note that all meaningful gestures presented in the study differed for hand/finger posture rather than arm posture. In a pilot experiment used as a pre-test, 12 participants were tested with various request, and meaningless gestures using the same procedure as in the present experiment (see below). The gestures chosen in the present study were those to which the participant responded successfully in all trials. In fact, the difficulty encountered by the participants in request gesture recognition was probably due to presentation of static postures. This did not occur for symbolic gestures whose meaning was immediately understood by the hand posture. Each actor's picture was presented simultaneously to a BEEP (duration of 0.25 s). The picture presentation of the conspecific lasted for 5.3 s. During the first 0.3 s the conspecific was presented in a rest posture [the hand(s) was/were placed on the table], whereas in the remaining 5 s he was presented after assuming the final posture. The interval between the presentations of two successive pictures was 5 s during which a blank panel was presented. The participants performed a go–no-go task. When a meaningful gesture was presented, they reached for, and picked up a red cylinder (diameter of 2 cm, height of 2 cm). This was placed on the table plane, 15 cm distant from SP, below the PC video. Specifically, the target was aligned with the actor's midline and its position was below the conspecific's chest. When a meaningless gesture was presented, the participant had to stay still. The participants were recommended to start to execute the action as soon as they recognized that the gesture was meaningful. Five repetitions for each meaningful gesture condition were presented in pseudo-random order (i.e., each gesture independently of unimanual or bimanual was presented ten times). Ten trials presented meaningless gestures. In total, one hundred trials were run divided into two blocks of 50 trials run in the same experimental session.

#### Data recording

The movements of the participants' right arm were recorded using the 3D-optoelectronic SMART system (BTS Bioengineering, Milano, Italy). This system consists of six video cameras detecting infrared reflecting markers (spheres of 5-mm diameter) at a sampling rate of 120 Hz. Spatial resolution of the system is 0.3 mm. The infrared reflective markers were attached to the nails of the participant's right thumb and index finger, and another marker was attached to the participant's right wrist. The markers attached to the thumb and index finger were used to analyze the grasp kinematics, whereas the marker attached to the wrist was used to analyse the kinematics of reaching. The data of the recorded movements were analyzed using a software developed using MATLAB version 7.7 (R2008b). Recorded data were filtered using a Gaussian low pass smoothing filter (sigma value, 0.93). The time course of reach-grasp was visually inspected: the beginning of the grasp was considered to be the first frame in which the distance between the two markers placed on the right finger tips increased more than 0.3 mm (spatial resolution of the recording system) with respect to the previous frame. The end of the grasp was the first frame after the beginning of finger closing in which the distance between the two right fingers decreased less than 0.3 mm with respect to the previous frame. The beginning of the reach was considered the first frame during which the displacement of the reach marker along any Cartesian body axis increased more than 0.3 mm with respect to the previous frame. To determine the end of the reach we calculated separately for the X, Y, and Z axes the first frame following movement onset in which the X, Y, and Z displacements of the reach marker decreased less than 0.3 mm compared to the previous frame. Then, the frame endpoint temporally closer to the grasp end frame was chosen as the end of the reach. The grasp was studied by analysing the time course of the distance between the index finger and thumb markers. From a pinch position, the grasp component is constituted by an initial phase of finger opening up to a maximum (maximal finger aperture) followed by a phase of finger closing on the object (Jeannerod, 1988). We measured the following grasp parameters: maximal finger aperture and parameters concerning the initial phase of finger opening which mainly reflect grasp planning; they were peak acceleration of finger opening, peak deceleration of finger opening, time to peak velocity of finger opening. Concerning the reach trajectory we analyzed reach peak velocity and, in addition, peak elevation (maximal height) of arm 3D trajectory and maximal curvature of arm 3D trajectory, that is the maximal distance in 3D space between the trajectory and the segment joining trajectory beginning and end. Both parameters allowed finding the gesture spatial effects on reach. Finally, we measured reach start (RS), that is the time to reach beginning with respect to trial beginning (beep presentation).

#### Data analysis

Repeated measures ANOVAs were carried out on the mean values of the reaching-grasping parameters and on RS. The within-subjects factors were type of gesture (request vs. symbolic), valence (positive vs. negative), and conspecific's gaze (gaze directed to the participant vs. not available gaze). In all analyses *post-hoc* comparisons were performed using the Newman–Keuls procedure. The significance level was fixed at *p* = 0.05.

### Experiment 2

#### Participants

A new sample of fourteen right-handed (Oldfield, 1971), naïve volunteers (7 females and 7 males, age 22–29 years) participated in the experiment. All of them were Italian native speakers.

#### Apparatus, stimuli, and procedure

The same apparatus, procedure, and stimuli as in Experiment 1 were used, except that the stimuli were pictures representing the conspecific in rest position, with their hands placed on the table. He was either blindfolded or directed his gaze straight ahead (i.e., toward the participant). The no-go response was required by pictures of the conspecific having assumed a meaningless posture as in Experiment 1. The go response, in contrast, was required when the conspecific assumed no hand/arm posture. Ten trials for each condition were presented in pseudo-random order. In total, 30 trials were run.

#### Data recording and analysis

Data recording was as in Experiment 1. Repeated measures ANOVAs were carried out on mean values of the reaching-grasping parameters and RS. The within-subjects factor was conspecific's gaze (gaze directed to the participant vs. not available gaze). The significance level was fixed at *p* = 0.05.

### Experiment 3

#### Participants

A new sample of 14 right-handed (Oldfield, 1971), naïve volunteers (7 females and 7 males, age 18–24 years) participated in the experiment. All of them were Italian native speakers.

#### Apparatus, stimuli, and procedure

**Figure 2:**
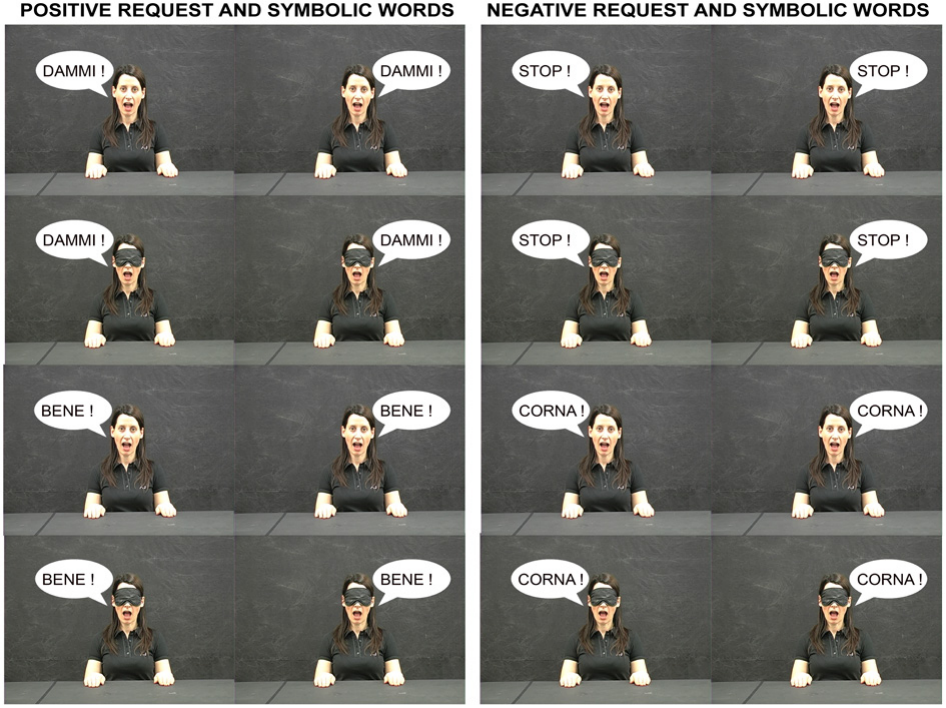
**Meaningful words shown by the conspecific (see the vignettes on the right and on the left) and presented in Experiment 3.** The conspecific could be either blindfolded or not. The upper two rows show request words and the lower two rows show symbolic words. The two columns on the left show words with positive valence whereas the two columns on the right show words with negative valence.

The apparatus and procedure were the same as in Experiment 1. The stimuli were pictures of the still conspecific: his hands were placed on the table plane and his mouth was open. The conspecific was blindfolded or not. In the second case he directed his gaze straight ahead. On a vignette placed either on the right or left conspecific's face one word corresponding-in-meaning to each of the gestures used in Experiment 1 was printed. The words “DAMMI” (give-me) and “STOP” were selected to be compared with the request gestures “give-me-in–the-hand” and “stop.” The words “BENE” and “CORNA” were selected to be compared with the symbolic gestures “well-done” and “horns” (Figure 2). We selected the word which better expressed the meaning of the gesture in a neutral context. For an example, the word “BENE” (well-done) better expresses the meaning of the gesture “thumb up” even if words such as “OK” “I AGREE” may be associated to the same gesture in a different context. The meaningful words were used as stimuli for the go condition in the task. Moreover, pseudo-words (i.e., “TANNI,” “VEBE,” “STOR,” and “GORMA”) were used as meaningless stimuli for the no-go condition of the task. Ten repetitions for each condition were presented in pseudo-random order (five with right vignette position and five with left vignette position in order to counterbalance in space the presented word as well as the bimanual gestures did). One hundred trials were run; they were divided into two blocks of 50 trials executed in the same experimental session.

#### Data recording and analysis

Data recording was the same as in Experiment 1. Repeated measures ANOVAs were carried out on the mean values of the reaching-grasping, and RS. The within-subjects factors were as follows: type of word (request vs. symbolic), word valence (positive vs. negative), and conspecific's gaze (gaze directed to the participant vs. not available gaze). In all analyses *post-hoc* comparisons were performed using the Newman–Keuls procedure. The significance level was fixed at *p* = 0.05.

## Results and discussions

### Experiment 1

Comparison between positive and negative request and symbolic gestures (“give-me-in-the-hand” vs. “stop” and “well-done” vs. “horns”). The conspecific was either blindfolded or his gaze was directed straight ahead (i.e., toward the observer/agent).

#### Grasp

The negative valence made finger opening faster. The request gesture slowed down finger opening.

**Figure 3:**
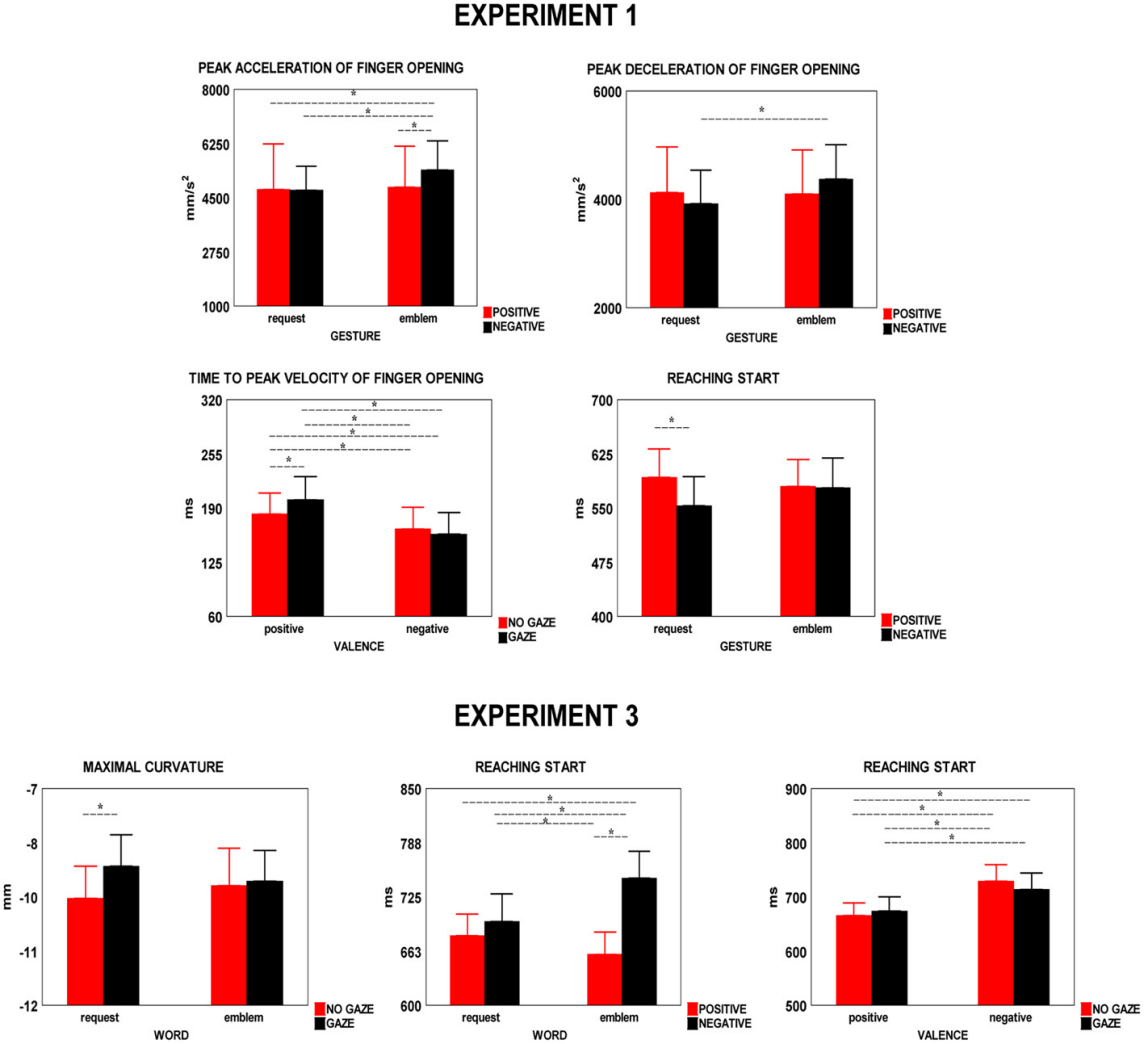
**Parameters of grasp, reach, and reaching start which were significant on ANOVA to the interaction between type of gesture (or word) and valence or valence and gaze in Experiments 1–3.** Vertical bars are SE. Asterisks indicate significance in *post-hoc* comparisons.

Specifically, *peak acceleration of finger opening* increased in presence of negative symbolic gestures (Table 1, Figure 3) in the comparison with the other conditions (*p* ≤ 0.03) which did not differ from each other (*p* ≥ 0.7). *Peak deceleration of finger opening* increased in presence of negative symbolic gestures as compared to negative request gesture (*p* = 0.05, Table 1, Figure 3). *Time to peak velocity of finger opening* decreased when the negative gesture (symbolic and request), both in not available (*p* = 0.026) and direct gaze (*p* = 0005) conditions, was presented, but this effect was greater in direct gaze condition. This parameter increased in presence of request gestures (Table 1, 184.8 vs. 168.3 ms).

**Table 1:**
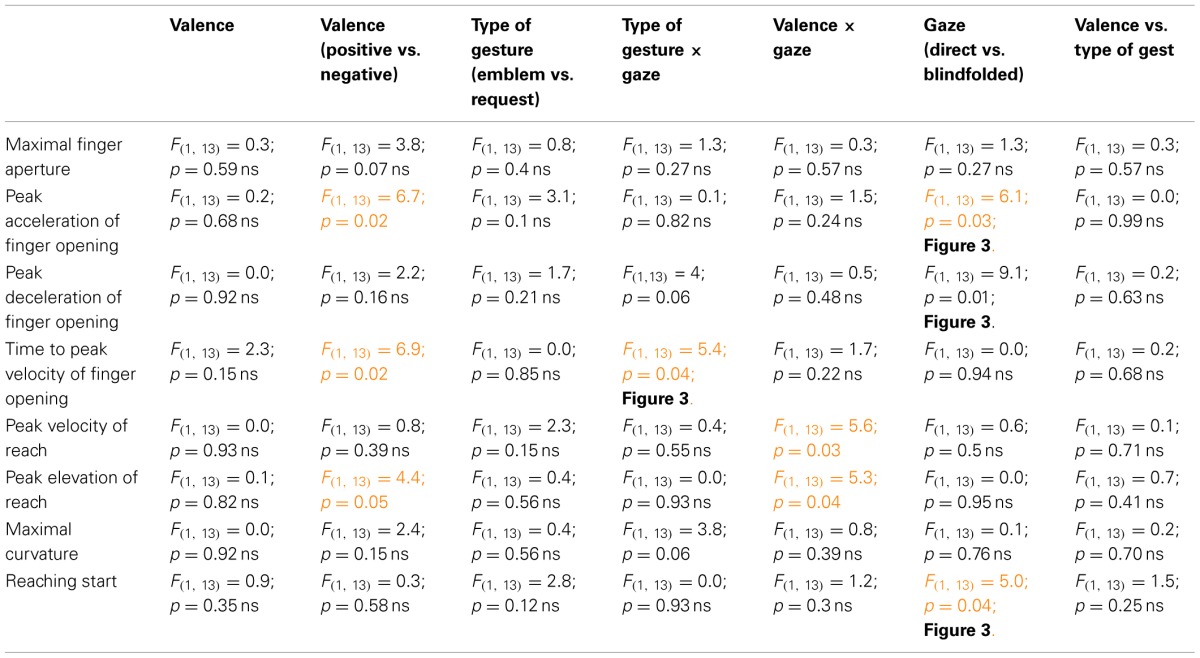
**Results of statistical analyses performed on kinematic parameters in Experiment 1**.

Red character indicates significance in ANOVA.

#### Reach

The requests made trajectory elevation lower. When the gaze was direct (available gaze), peak velocity, and trajectory elevation decreased.

Specifically, *peak elevation* of reach was lower in presence of requests (Table 1, 97 vs. 98 mm) and when the conspecific's gaze was direct (Table 1, 96 vs. 98 mm). *Reach peak velocity* decreased in the condition of conspecific's direct gaze (Table 1, 426 vs. 431 mm/s).

#### Reach start

*RS* was longer when the request was positive (i.e., “give-me-in-the-hand”) as compared to the negative request (Table 1, Figure 3, *p* = 0.02).

Experiment 1 leaves unsolved the issue of whether effects of different gazes (direct vs. not available) can be observed even when variations in gaze are not accompanied by gesture presentation.

### Experiment 2

Effects of the sole gaze on the action: comparison between not available gaze and conspecific's direct gaze.

#### Reach start

*RS* increased when the gaze was directed to the agent (Table 2, 878 vs. 805 ms).

**Table 2:**
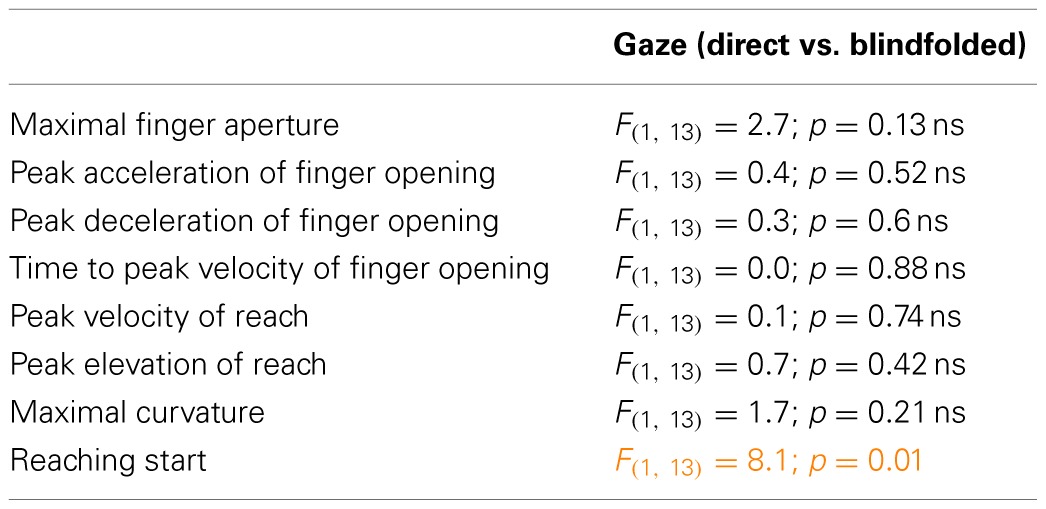
**Results of statistical analyses performed on kinematic parameters in Experiment 2**.

Red character indicates significance in ANOVA.

In Experiment 1, when the conspecific produced gestures, the conspecific's gaze directly affected the observer's behavior and, in addition, modulated the response to other gesture characteristics, when these were effective. In contrast, in contro Experiment 2, when no gesture was presented, it did not directly intervene in action kinematics, i.e., it did not assume the meaning of a message aimed at changing the behavior of the observer. The increase in RS due to direct gaze, which was absent in Experiment 1, may be consequent to the time lost by the observer in waiting for a possible gesture from the conspecific since fixating a conspecific usually accompanies gesture or word production.

Experiments 1–2 showed that type of gesture, valence, and gaze influenced the execution of actions. It has been shown that gesture and word are functionally related to each other (Bernardis and Gentilucci, 2006; Gentilucci et al., 2006, 2008; Gentilucci and Dalla Volta, 2008; Barbieri et al., 2009; Gentilucci and Campione, 2011). Consequently, the same characteristics of words could influence action kinematics as the corresponding gestures.

### Experiment 3

Comparison between words corresponding in meaning to request and symbolic gestures presented in Experiment 1 by blindfolded and not blindfolded conspecific (give-me vs. stop and well vs. horns).

The word valence did not affect the action kinematics, whereas type of word and gaze did. The request gestures increased maximal finger aperture and, with the gaze, trajectory curvature of reach.

#### Grasp

*Maximal finger aperture* was greater in presence of request words (Table 3, 84 vs. 83 mm).

**Table 3:**
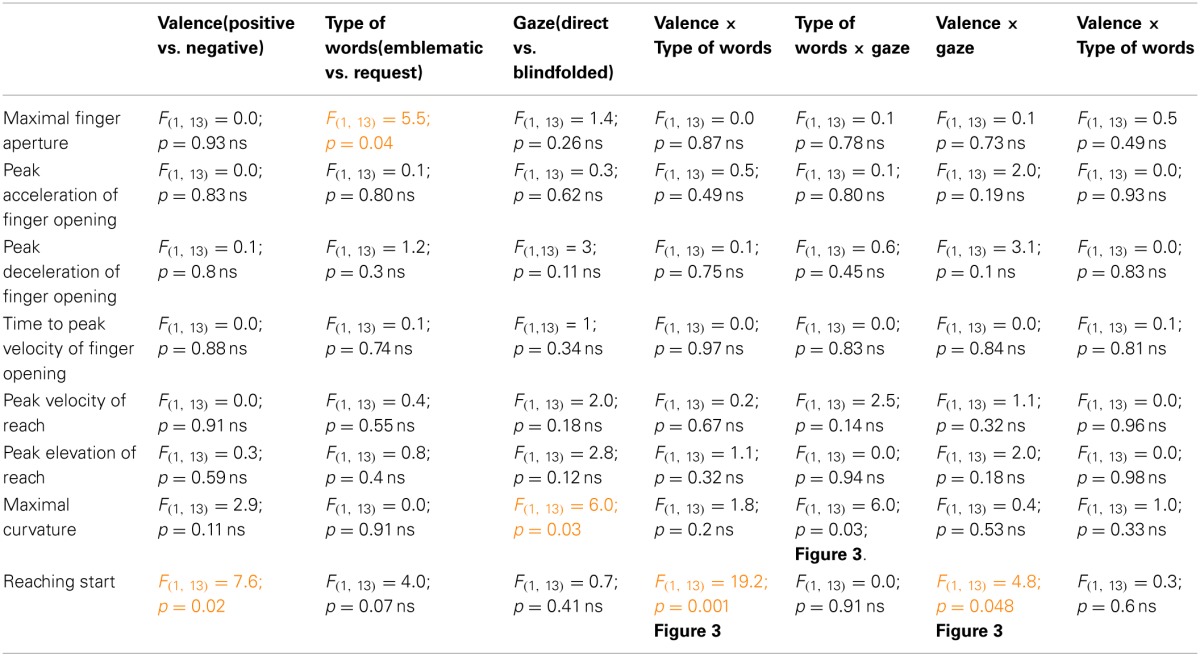
**Results of statistical analyses performed on kinematic parameters in Experiment 3**.

Red character indicates significance in ANOVA.

#### Reach

*Maximal curvature* of reach trajectory increased with conspecific's direct gaze as compared to blindfolded conspecific. It occurred when a request word was presented. (Table 3, Figure 3, *p* = 0.04). No other significance in *post-hoc* comparisons was found (*p* ≥ 0.19).

#### Reach start

*RS* increased when the symbolic word was negative as compared to the other conditions (Table 3, Figure 3, *p* ≤ 0.017). No statistical difference was found between negative and positive requests (*p* = 0.19). Finally, concerning the interaction between valence and gaze (Table 3, Figure 3) no significance was found between negative (*p* = 0.07) and positive (*p* = 0.29) words in both conditions of blindfolded and not blindfolded conspecific.

## General discussion

The three characteristics of the gestures analyzed in the present study that is type of gesture, valence, and gaze, produced by a conspecific influenced the behavior of an observer/agent. The influence was observed when no actual interaction was possible between observer and gesturing conspecific because the gestures were presented as pictures in a PC video. Thus, the effects found on the action (reaching-grasping) kinematics can be attributed to automatic observer's response to the gesture independently of the physical presence of a gesturing individual. They exclude an increase in accuracy in movement control, due to the possibility that the conspecific if physically present could directly intervene in action execution. In addition, these effects depended neither on automatic imitation of arm/hand kinematics because postures were only presented, nor on position of the arm because the upper arm postures were similar for all gestures; what changed was the hand/finger posture.

The type of gesture had a different impact on the action: the request gestures induced a slowing down of the initial grasp and a lowering of reach trajectories as compared to symbolic gestures. We interpreted these differences between the two types of gesture as due to the request gestures which are more impelling in requiring an immediate and stronger response (Barten, 1979) as compared to symbolic gestures. Note that the two requests could induced opposite observer's behaviors (e.g., approaching or avoidance of the conspecific) which, however, were both different from the actual action directed to the target-cylinder. These behaviors could interfere slowing down the initial grasp and could be compensated producing lower trajectory toward the target. Interference was present or greater for gestures requiring stronger and more immediate response. Note that lower trajectories indicate veering away from the conspecific's gesturing hand. This result is in accordance with results of studies in which visual distractors were presented together with a target (Tipper et al., 1991, 1997; De Stefani et al., 2012) or a gesture of pouring request was presented to an observer (Innocenti et al., 2012). In both conditions, the hand veered away from the distractor object or the gesturing hand.

The negative as compared to the positive valence affected grasping, whereas the positive request “give-me-in-the-hand” influenced RS. The negative valence affected different grasp kinematic parameters, Taken together these effects made initial grasping quicker. This result can be explained as follows: the participants executed the action or part of it more quickly in the case of conspecific's threatening attitude related to the negative communication, at least till they took possession of the target (this explains why it affected grasping rather than reaching). In fact, the threatening attitude could induce competition when the participants had to perform a movement in the space close to the gesturing conspecific: this trend to competition could make faster the actual action (Georgiou et al., 2007). Note that in the present study the threatening attitude seemed to be less effective to induce competition for requests than for symbolic gestures. In fact, “stop” may be produced even with a collaborative intent. Obviously, this does not occur for “horns.”

The effect of the positive request on RS could depend on the fact that this gesture (“give-me-in-the-hand”) required an interaction with a specific body part (the conspecific's hand) and probably its spatial localization when planning a program compatible with the gesture (i.e., before movement beginning) interfered with the actual target localization.

Variation in gaze alone did not affect the action kinematics in Experiment 2 when no gesture was presented, whereas, in Experiment 1, when gestures were presented, different gazes directly affected movement and, in addition, modulated the effectiveness of the gesture characteristics. Usually, direct gaze independently of gesture slowed down movement because it produced interference. In addition, it interacted with gesture characteristics increasing their effects. Note that eye contact means that a communication is directed just to the observer (Allison et al., 2000; George and Conty, 2008; Senju and Johnson, 2009). In contrast, when a blindfolded gesturing conspecific was presented, observer remained uncertain if the gesture was directed to him/her self. Consequently, direct gaze made more effective the response to the gesture and produced greater interference. These data are in accordance with the results of previous studies in which the effects of gaze availability on requests were studied (Sartori et al., 2009; Ferri et al., 2011; Innocenti et al., 2012). In Innocenti et al.'s study (2012), gaze alone without presentation of request made grasp execution quicker: this finding was interpreted either as a request of quick performance since no hand request was produced or it induced embarrassment in the participant. It was likely that this was absent in the present study, because we presented a picture of the conspecific, whereas in Innocenti et al.'s study (2012) the conspecific was physically present. In fact, the physical presence of an individual may be more effective for communicating messages by eyes.

In an fMRI study, Ferri et al. (unpublished data) found a wide right-lateralized network comprising prefrontal cortex, inferior frontal gyrus, ventral premotor cortex, pre-supplementary motor area, inferior parietal lobule, superior and middle/inferior temporal gyri more strongly activated by request gestures performed by blindfolded as opposed to not-blindfolded conspecific. This likely reflected the effort to fully understand the conspecific's potential interest for interacting, when relevant social cues, as direct gaze or spoken language, were missing. The data of the present study suggest that because of this uncertainty about the conspecific's intention to interact, weaker response to the gesture was activated.

Finally, we aimed at determining whether the same characteristics affected the kinematics of the action when words corresponding in meaning to the gestures of the Experiment 1 were presented. In general, the effects of the words were less evident than those of the gestures, except type of word. Type of word affected reaching and grasping, whereas gaze affected reaching. Word valence did not affect the action kinematics.

The request words in comparison with symbolic words caused an increase in maximal finger aperture. We explain the increase in maximal finger aperture as compensation for uncertainty (producing an increase in arm trajectory variability) during reaching-grasping of the actual target (Chieffi and Gentilucci, 1993). This was due to activation of a more interfering program in response to the request, which competed with the actual action. Compensation of request word was more complex than compensation of request gesture: the former included the relations between grasp and reach and, consequently, the whole action of reaching-grasping, whereas the latter was an interference which acted on the initial part of grasp only. Consequently, it seems that the requests expressed by words were stronger because they required more compensation than gestures. In addition, request word and direct gaze also affected reach trajectories which veered away from the “speaking” conspecific. Note that the participants produced more curve trajectories in order to avoid the conspecific's body, whereas when presenting gestures lower trajectories were produced in order to avoid the conspecific's hand.

Sartori et al. (2009) found that request gestures induced a deviation of arm trajectory toward the requiring conspecific. This result contrasts with that of the present study, where both gestures and words induced trajectory deviation away from the requiring conspecific. However, it can be explained by the fact that those authors presented a sudden request during movement execution, whereas we presented a request before movement initiation. Consequently, in the former case the request might have produced an assimilative effect, whereas in the latter case it might have produced a contrastive effect. This might have been consequent to time at disposal for elaboration of a response. Another possibility is that we presented pictures of the gestures, whereas Sartori et al. (2009) showed a physically present conspecific. Against this possibility Ferri et al. (2011) found the same results as ours in a study in which the gesturing conspecific was physically present.

Word valence affected RS. The negative emblem increased RS; this finding probably depended on the scarce communicative aspect of the word “CORNA” (horns) as compared to the corresponding gesture. In other words, the increase in RS could be consequent to the difficulty to associate the word to the message of the conspecific to not interact. The fact that no effect of the positive request was found on RS as in gesture experiment may be due to the positive request word (“DAMMI,” give me) whose meaning is more abstract (or less specified) than the corresponding gesture (“give-me-in-the-hand”). Indeed, the gesture implied interaction with a specific conspecific's body part, acting in Experiment 1 as a distractor during localization of the action target.

Effects of the gesture valence were found on kinematic parameters collected in Experiment 1 rather than word valence in Experiment 3, probably because the gestures are more related than words to emotional aspects in the interactions between conspecifics. That is, the gesture can transmit more easily the attitude related to the valence. Note also that in Experiment 3 printed words were presented (and the actor was inexpressive), instead of an actor pronouncing words aloud. It is well-known that attitudinal aspects of spoken language can be mainly expressed by variation in prosody obviously not modulated in the present experiment.

The result that gesture characteristics, such as valence, influenced actual behavior more than speech, may also depend on a stronger link between the motor program of the gesture automatically activated by observation in order to understand the gesture meaning and the motor program activated for a response compatible with the gesture meaning. In support of this hypothesis, Newman-Norlund et al. (2007) found the same circuit (MNc, Mirror Neurons circuit) activated by both imitation of gestures and activation of the motor programs in response to the same gestures.

However, previous studies found that word valence (positive/negative) influenced movements (Chen and Bargh, 1999; Freina et al., 2009). Their authors suggested that positive words are connected to approaching movements, whereas negative words are attached to avoidance tendencies. Thus, positive words should induce faster approach of the target as compared to negative words. This was not found in the present study. This may be explained by the neutral word aspect, i.e., by the lack of attitudinal and emotional aspects for the presented words which solicited a specific motor response to the word meaning rather than to the attitude it evoked (see above). For an example, the word “DAMMI” (give-me) can be interpreted as a peremptory order independently of agent's will (negative aspect) as well as a request of constructive cooperation (positive aspect). However, negative/positive word aspect was not specified when intonation was lacking. In addition, the gesture valence induced an effect opposite to that found for word valence (Chen and Bargh, 1999; Freina et al., 2009). However, considering the gestures we presented, the valence probably induced an attitude to compete (negative valence) or to cooperate (positive valence). Competition as compared to cooperation usually makes faster the movement.

In summary, gestures affected the control of an action apparently unrelated to gesture meaning. Type of gesture, valence, and gaze contributed to modulate these effects. Similar effects were found for communicative words even if valence was not effective and the responses to the other characteristics differed even if the general effect on the action was similar. We have proposed that the responses to these communicative signals are not independent of the type of signal (either gesture or word) but probably both of them concur to an integrated and, consequently, more effective response of the observer when they are simultaneously produced.

### Conflict of interest statement

The authors declare that the research was conducted in the absence of any commercial or financial relationships that could be construed as a potential conflict of interest.
